# HLA不合造血干细胞移植患者供者特异性抗HLA抗体检测结果临床解读中国专家共识（2025年版）

**DOI:** 10.3760/cma.j.cn121090-20250725-00348

**Published:** 2025-10

**Authors:** 中华医学会血液学分会干细胞应用学组

## Abstract

供者特异性抗HLA抗体（DSA）导致的HLA不合异基因造血干细胞移植后原发性植入失败仍是患者死亡的重要原因之一。近年来，针对DSA与HLA不合移植预后的关系以及DSA去敏治疗等方面的研究取得了显著进展。在此基础上，中华医学会血液学分会干细胞应用学组组织相关专家制定了本共识，旨在更好地指导DSA阳性HLA不合移植候选患者去敏治疗的临床实践。

## Abstract (en)

Donor-specific anti-HLA antibodies (DSA)-induced primary graft failure remains a significant cause of mortality in patients undergoing HLA-mismatched allogeneic hematopoietic stem cell transplantation. In recent years, significant advances have been made in understanding the association between DSA and prognosis following HLA-mismatched transplantation, as well as in the development of DSA desensitization therapies. Based on these advances, the Hematopoietic Stem Cell Application Group, Chinese Society of Hematology, Chinese Medical Association has developed this consensus to better guide the clinical practice of desensitization therapy for DSA-positive, HLA-mismatched transplant candidates.

异基因造血干细胞移植（allo-HSCT）是恶性血液病和非恶性血液病有效、乃至唯一的治愈手段[1]–[3]。过去二十年中，单倍型相合造血干细胞移植（haplo-HSCT）体系的建立和普及使得“人人都有移植供者”从理想变为现实[2]–[4]。目前，欧洲、美国和我国接受haplo-HSCT患者占接受allo-HSCT患者的比例分别为20.3％、25.0％和52.7％；欧美及我国的统计数据显示，HLA不合其他供者已经成为主要供者来源之一[1],[5]–[6]。对于接受HLA不合移植的患者而言，供者特异性抗HLA抗体（DSA）导致的原发性植入失败（pGF）［包括原发性移植排斥（GR）和原发性植入不良（PGF）］仍是造血重建成功的主要障碍和死亡的重要原因之一[7]–[12]。

近年来，国内外学者在移植候选患者体内DSA检测、去敏治疗等方面的研究取得系列成果，使DSA导致的pGF发生率显著降低[11],[13]–[18]。目前，我国尚缺乏针对allo-HSCT患者DSA检测以及如何将DSA检测结果应用于临床实践的共识。为推动DSA检测在HLA不合移植中的规范化应用、提高移植疗效，中华医学会血液学分会干细胞应用学组组织国内移植领域和血液病诊断领域的相关专家，结合国内外研究进展，讨论制定了本共识。

一、DSA的概念和HLA抗体检测方法

DSA指移植候选患者体内预先存在的抗体，这些抗体可针对供者不匹配的Ⅰ类（HLA-A、HLA-B、HLA-C）和（或）Ⅱ类（HLA-DRB1、HLA-DQB1、HLA-DP）等抗原产生免疫反应[19]。抗HLA抗体检测方法见《异基因造血干细胞移植中抗HLA抗体检测质量控制、结果分析和报告中国专家共识（2025年版）》[20]。

二、候选allo-HSCT患者抗HLA抗体和DSA的检出率以及危险因素

健康男性和女性体内抗HLA抗体的阳性率分别为1％和17.3％；有妊娠史的健康女性体内抗HLA抗体的阳性率为24.4％，妊娠超过3次的健康女性体内抗HLA抗体的阳性率可达32.2％[21]–[23]。haplo-HSCT候选患者体内抗HLA抗体和DSA的阳性率分别为20％～70％和10％～30％[8],[13]–[14],[24]；无关供者移植或脐血移植候选患者体内抗HLA抗体和DSA的阳性率分别为20％～40％和1％～7％[25]–[27]。移植候选患者体内产生抗HLA抗体和DSA的高危因素包括女性、高龄、多次怀孕史以及多次输血史（尤其是输注血小板）[16],[25]–[27]。

**专家组推荐：**临床工作中，移植医师应重点关注具有产生抗HLA抗体和DSA高危因素的移植候选患者。

三、DSA、非DSA和非抗HLA抗体对移植患者预后的影响

移植前DSA阳性但未经去敏治疗的HLA不合无关供者移植和脐血移植患者接受移植后中性粒细胞植入和血小板植入较DSA阴性患者明显延迟[28]–[29]。无论患者接受haplo-HSCT、HLA不合无关供者移植还是脐血移植，移植前DSA阳性都是导致移植后pGF的主要影响因素之一[28]–[30]。最近的两项荟萃分析显示，移植前DSA阳性患者移植后pGF的发生率是DSA阴性患者的7.47倍（*P*<0.001）和6.82倍（*P*<0.001）[31]–[32]。尽管多数研究证实，移植前DSA阳性与pGF密切相关，但不同移植中心确定的DSA阳性平均荧光强度（MFI）阈值包括1 000、5 000、10 000等。文献中报道的DSA针对HLA抗原的位点包括A、B、C、DRB1、DQB1以及DPB1等[31]–[32]。此外，北京大学血液病研究所团队还发现，移植前DSA MFI≥2 000的患者接受haplo-HSCT后PGF的发生率显著高于移植前DSA MFI<2 000的患者（*P*＝0.005）[30]。总之，美国和我国学者发现，移植前DSA MFI<2 000的患者接受haplo-HSCT后GR或PGF的发生率与DSA阴性患者无差异；移植前DSA MFI≥2 000的患者GR或PGF的发生率随抗体滴度的升高而增加[30]。

对于接受基于移植后环磷酰胺诱导免疫耐受的haplo-HSCT患者而言，移植前DSA MFI>20 000或去敏治疗后C1q阳性与移植后非复发死亡率（NRM）高、无进展生存（PFS）率低、总生存（OS）率低密切相关[33]。基于粒细胞集落刺激因子诱导免疫耐受的haplo-HSCT模式下，移植前DSA MFI≥ 10 000是患者NRM高、PFS率低、OS率低的危险因素。荟萃分析显示，DSA是移植后PFS差（haplo-HSCT，*HR*＝3.19，*P*<0.001）和OS差（脐血移植，*HR*＝1.68，*P*＝0.030）的危险因素[31]–[32]。

非DSA与移植预后的关系尚存争议，Takanashi等[27]的研究显示，非DSA可能与中性粒细胞植入率降低相关。然而，其他学者未观察到非DSA对植入等移植预后的不良影响[25],[34]。除了抗HLA抗体以外，在移植候选患者体内还可检测到抗主要组织相容性复合体Ⅰ类相关链A（MICA）抗体。尽管有研究显示，MICA与脐血移植后的血小板植入率低有关[35]，但MICA与移植预后不良的相关性尚缺乏足够证据。

**专家组推荐：**

①HLA不合移植前应常规检测抗HLA抗体，并通过比对供受者HLA配型结果，评判移植候选患者体内是否存在DSA；

②未来应开展多中心研究观察非DSA以及MICA等对移植预后的影响。

四、DSA去敏治疗

目前，DSA去敏治疗方法包括：①利用血浆置换（PE）或免疫吸附降低移植候选患者血液循环中的抗体水平；②利用人血免疫球蛋白中和抗体；③利用抗CD20单克隆抗体或蛋白酶体抑制剂分别清除B细胞或浆细胞、降低抗体水平；④利用供者来源的抗原（辐照的HLA不合供者血小板）阻断DSA通过抗体依赖细胞介导的细胞毒作用（ADCC）或补体依赖的细胞毒性（CDC）途径介导的造血干/祖细胞的损伤；⑤阻断补体级联反应[9]–[10],[33],[36]–[42]。

由于约45％的DSA分布在血液循环中，所以DSA可在PE后1～2周恢复到基线水平；此外，应用PE大量清除DSA后会导致抗体反跳至基线水平以上[43]。因此，PE后应该桥接其他去敏治疗和（或）预处理，随后进行移植以避免抗体反跳导致的去敏治疗效果降低或失败。

美国MD安德森癌症中心的回顾性分析显示，4例患者接受PE联合利妥昔单抗治疗，移植后2例获得造血重建。中国医学科学院血液病医院的回顾性研究显示，7例回输供者血小板联合利妥昔单抗进行去敏治疗的患者接受haplo-HSCT后DSA水平显著降低，所有患者均获得植入[44]。迄今为止，大多数国内外关于去敏治疗的研究为回顾性研究（见表1）[33],[44]–[50]，这些研究中的去敏治疗方案为2种、3种、4种，甚至5种方案联合。北京大学血液病研究所开展的一项前瞻性研究纳入55例接受haplo-HSCT的患者，这些患者移植前的DSA MFI水平为2 000～10 000，均于移植前3 d（−3 d）接受单次剂量利妥昔单抗（375 mg/m^2^）治疗，结果显示，94.5％的患者获得造血植入，移植前DSA MFI 5 000～10 000的患者移植后PGF发生率显著高于移植前DSA MFI 2 000～5 000的患者（13％对0，*P*＝0.037）[9]。该中心随后的大样本研究显示，对于接受单次剂量利妥昔单抗去敏治疗的患者而言，移植前DSA MFI>5 000是移植后发生PGF的高危因素，提示对于这些患者有必要采用联合去敏治疗方案[38]。

**表1:** DSA阳性HLA不合异基因造血干细胞移植患者的去敏治疗方法

去敏治疗方法的分类	目前常用及处于临床前和临床研究中的去敏方法
去除血液循环中DSA	血浆置换术或免疫吸附疗法，如双重滤过血浆置换术
抗体中和/增强DSA清除	人血免疫球蛋白；供者血小板输注
靶向B细胞	靶向CD19的CAR-T细胞^a^；针对CD20单克隆抗体，针对CD19或CD20的双特异性抗体^a^
靶向浆细胞	靶向CD38和（或）BCMA的CAR-T细胞^a^；针对CD38的单克隆抗体^a^，蛋白酶体抑制剂
靶向Tfh	西罗莫司、针对白细胞介素-6的奥洛珠单抗等^a^
补体系统抑制剂	靶向C5a的单克隆抗体^b^，人血免疫球蛋白

**注** DSA：供者特异性抗HLA抗体；Tfh：滤泡辅助性T细胞；CAR-T细胞：嵌合抗原受体T细胞；^a^需要更多的临床试验证实疗法的有效性和安全性；^b^靶向C5a的单克隆抗体未曾用于异基因造血干细胞移植患者，需要开展探索性临床试验

浙江大学团队利用双重PE联合利妥昔单抗对33例移植前DSA阳性的haplo-HSCT患者进行去敏治疗，结果显示，所有患者均获得造血植入，去敏治疗前后DSA的中位MFI分别为7 505和2 013（*P*<0.001）。研究者还发现，经过去敏治疗的DSA阳性患者和同期DSA阴性患者移植的预后无差别[45]。Hashem等[51]采用欧洲血液和骨髓移植学会推荐的去敏治疗方法［包括静脉注射免疫球蛋白（IVIG）、利妥昔单抗和PE］治疗移植前DSA MFI>8 000的患者；对于移植前DSA MFI>3 000且<8 000的患者，仅予IVIG联合利妥昔单抗治疗。对于移植前DSA MFI 1 000～5 000的患者，La Rocca等[39]予患者利妥昔单抗联合IVIG去敏治疗，结果显示3例患者均获得造血植入。

最近，北京大学血液病研究所团队的一项研究显示，DSA阳性患者体内的滤泡辅助性T细胞（Tfh）存在数量和功能异常[36]。该团队的探索性研究显示，若以去敏治疗后DSA水平降低50％作为疗效评价指标，单独应用针对Tfh的西罗莫司、单用利妥昔单抗及二者联合应用的有效率分别为43.75％、30％和60％[36]。对15例接受西罗莫司单药（9例）或西罗莫司联合利妥昔单抗（6例）去敏治疗有效患者移植的预后进行分析，结果显示，所有患者均获得造血重建，预期1年PFS率和OS率均为100％[52]。此外，硼替佐米、抗CD38单抗以及嵌合抗原受体T细胞也被用于DSA去敏治疗[15],[53]（表1）。

总之，国内外研究提示，对于HLA-A、B、C、DR或HLA-DP、DQ移植前DSA阳性患者，均应予去敏治疗。北京大学血液病研究所团队的研究显示，去敏治疗的DSA MFI阈值为2 000[30]。

**专家组推荐：**

①对haplo-HSCT前DSA阳性（MFI<5 000且≥2 000）、无供者可替换的患者进行去敏治疗（单药利妥昔单抗、西罗莫司或与IgG联合等）；

②对于haplo-HSCT前DSA阳性（MFI≥5 000）、无供者可替代且急需移植的患者，应联合应用多种方法（PE、利妥昔单抗和IgG等）进行去敏治疗；

③对于去敏治疗无效的患者，建议进入临床试验探索新的去敏治疗方案。

五、DSA检测的时机和频率

移植前DSA阳性患者去敏治疗后再次评估抗体水平的时间点在不同研究中存在较大差异，尚缺乏共识。部分研究将评估去敏治疗效果的时间点定在完成去敏治疗后或−1 d[37]。Leffell等[54]将去敏治疗后DSA水平评估的时间点定在−7 d和−1 d，结果显示，13.3％的患者在−1 d时DSA水平再次升高，这些患者接受PE联合免疫球蛋白治疗后DSA转阴。北京大学血液病研究所团队Chang等[9]对28例接受去敏治疗患者移植后的DSA水平进行了动态观察，发现haplo-HSCT后7 d DSA MFI>1 000的患者原发性PGF的发生率显著高于MFI<1 000的患者（33％对0，*P*＝0.040）。一些研究显示，在去敏治疗后每周评估1次DSA或造血干细胞回输后1个月评估DSA或造血植入前每周评估1次抗体滴度[9],[40],[47]。

**专家组推荐：**

①去敏治疗过程中、去敏治疗后及造血干细胞回输前后有必要测定DSA水平，以确定抗体是否被清除；

②干细胞回输后测定DSA水平的时间点和频率尚不清楚，有条件的中心可每周评估1次直至造血植入和（或）DSA MFI<1 000；

③对于移植后发生GR或PGF的患者，建议尽快采集标本测定DSA水平；

④目前尚缺乏关于DSA检测时机和频率的循证医学证据，在临床实践中，建议移植医师在参考本共识的基础上，根据患者个体情况灵活调整DSA监测频率。

六、小结

目前，HLA不合移植前对患者体内DSA水平进行检测，并对DSA阳性患者更换供者或进行去敏治疗已成为HLA不合allo-HSCT的临床常规流程[9],[38],[45]–[46]（图1）。在获得高水平临床证据之前，各移植中心可以参考本共识推荐，结合移植前DSA抗体滴度、移植类型、去敏药物的作用机制等选择去敏治疗方案。对于接受去敏治疗的患者，应注意去敏治疗后抗体滴度下降的水平，并根据DSA水平对治疗方案进行必要的调整，以达到有效去敏、促进造血植入的目的。

**图1:**
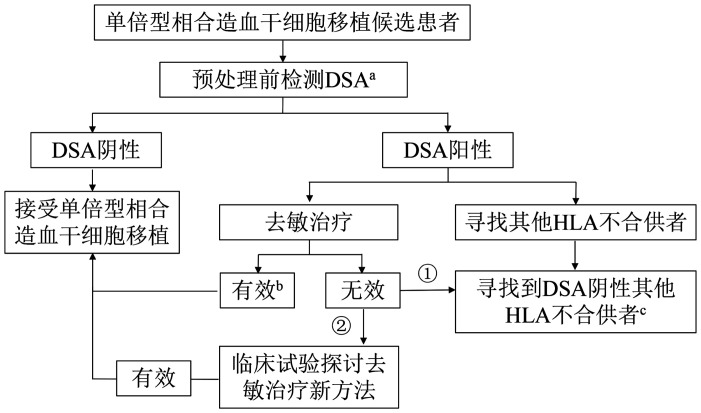
HLA不合造血干细胞移植候选患者DSA去敏治疗及供者选择建议 **注** HLA：人类白细胞抗原；DSA：供者特异性抗HLA抗体；^a^建议在预处理前6周进行DSA检测，为去敏治疗提供4～5周时间。^b^目前文献中尚无去敏治疗有效的统一定义，本共识中有效定义为：（1）完全有效：DSA阳性移植候选患者经去敏治疗后平均荧光强度（MFI）较治疗前降低超过50％，具体MFI值降至2 000以下[50]；（2）部分有效：DSA阳性移植候选患者经过去敏治疗后MFI较治疗前降低超过50％，具体MFI值降至5 000以下[36]。去敏治疗后MFI<2 000的患者可直接进行移植；对于治疗后MFI<5 000，但仍>2 000的患者，建议预处理第3天予利妥昔单抗375 mg/m^2^，0 d回输1份DSA阴性的脐血[9]。^c^对于以无关供者移植或脐血移植为特色的中心，也可参考该建议执行，仅将“单倍型相合造血干细胞移植候选患者”更换为“无关供者移植或脐血移植候选患者”。①对于拟选择单倍型相合供者，但现有方法去敏治疗无效，且有DSA阴性无关供者或脐血的患者而言，可选择无关供者或脐血。②对于拟选择单倍型相合造血干细胞移植，但现有方法去敏治疗无效，且缺乏DSA阴性无关供者或脐血的患者而言，可进入临床试验接受新方法去敏治疗
